# Knowledge tests on adolescent depression and anxiety: a measure for adults in the Chilean school system

**DOI:** 10.1186/s40359-026-04674-4

**Published:** 2026-06-20

**Authors:** Javiera Jara-Jara, Mónica Bravo-Sanzana, Leomari Mendoza-Caripa, Oscar Terán-Mendoza

**Affiliations:** 1 Universidad de La Frontera, Temuco, Chile; 2 Observatorio de Ciudadanía, Convivencia y Bienestar Educativo, Temuco, Chile; 3 Departamento de Psicología, Facultad de Ciencias de la Salud, Universidad Católica de Temuco, Temuco, Chile

**Keywords:** Depression, Anxiety, Adolescence, School, Mental health, Psychometrics

## Abstract

Concerns about the mental health of young people have increased in recent years, and the knowledge of education professionals about mental health disorders is closely related to the identification of young people with these problems. Due to the scarcity of instruments to measure this knowledge, the present research aimed to develop and validate tests to measure knowledge about adolescent depression and anxiety to be answered by adults in the school system. We used a quantitative, non-experimental, and cross-sectional design. A non-probabilistic purposive sampling method was used to select participants. The sample comprised 10 expert judges for content validation, 16 teachers for cognitive interviews, and 304 professionals from educational establishments for administering the tests. Items were developed based on a literature review and the DSM-5; concordance among judges was calculated using Aiken’s V coefficient. Difficulty and discrimination analyses were carried out using Classical Test Theory (CTT), a well-established framework that is well-suited to the sample size and the exploratory nature of this study. Group contrast was used as validity evidence, and the Kuder-Richardson reliability coefficient was calculated. In total, 45 items resulted; the depression test has 17 items in the symptoms dimension and 10 in risk factors, and the anxiety test has 12 in the symptoms dimension and 6 in risk factors. In terms of reliability, the depression test yielded coefficients of 0.864 for the symptoms dimension and 0.742 for the risk factors dimension. The anxiety scale showed coefficients of 0.873 and 0.846 for the symptoms and risk factors dimensions, respectively. The results provide preliminary psychometric evidence of content validity and internal consistency, based on a non-probabilistic regional sample from southern Chile. Future research should replicate these findings in larger, more diverse samples and apply advanced modeling approaches, such as Item Response Theory, to further evaluate the instruments’ properties.

**Supplementary Information:**

The online version contains supplementary material available at 10.1186/s40359-026-04674-4.

## Introduction

Mental health problems in young people have been increasing considerably over the years, mainly due to the pandemic context and the confinement measures that were taken [1]. Addressing these problems effectively requires that adults who interact regularly with adolescents, particularly those in educational settings, recognize early signs and respond appropriately [2]. Mental health literacy offers a framework for understanding how adults in community settings can contribute to early intervention. Jorm et al. [3] introduced this term to refer to beliefs and knowledge about mental disorders that aid their recognition, management, and prevention. Mental health literacy encompasses multiple distinct components, including attitudes toward help-seeking, knowledge of treatments and symptoms, and practical help-seeking skills, as well as knowledge of risk factors [4]. This study isolates and operationalizes this latter component, developing instruments that assess specific, actionable knowledge regarding symptoms and risk factors of adolescent depression and anxiety, distinct from the broader attitudinal and behavioral dimensions of literacy.

In this regard, it is important to distinguish between instruments that measure a specific type of mental health knowledge, such as those developed in this study, and those designed to measure the construct of mental health literacy, as these scales serve different purposes and target different populations. Regarding mental health literacy, one of the most established scales is the Mental Health Literacy Scale (MHLS), created by O’Connor & Casey [5]. This instrument is a 35-item unidimensional measure that operationalizes the six dimensions of mental health literacy proposed by Jorm et al. [3]; it is designed to be completed by adults in the general population regarding their own psychological functioning. In the case of the educational context, a recent systematic review documented marked heterogeneity in the instruments used to assess teachers’ mental health literacy, with a predominance of non-standardized or ad hoc-adapted questionnaires, and disproportionate coverage of externalizing disorders such as ADHD, to the detriment of internalizing disorders such as depression and anxiety [6].

In the same vein, the tests developed in this study differ from the instrument created by O’Connor & Casey [5] in two respects: (a) they focus exclusively on the subcomponent of knowledge regarding symptoms and risk factors, in line with the expected preventive and non-clinical role of educational professionals; (b) they are specifically aimed at adults within the school system as informants regarding a third party (the adolescent student) rather than their own experience; while they differ from those found in the review by Johnson et al. [6] in that they are designed for high-prevalence internalizing disorders in adolescents, thereby addressing a documented gap in the literature.

Knowledge of symptoms and risk factors is directly linked to school-based mental health practices. Gatekeeper-based approaches to suicide prevention and mental health promotion consistently identify the recognition of symptoms and warning signs as a necessary prerequisite for early identification of risk situations and timely referral to specialized services [7, 8]. This recognition-to-referral pathway is well documented in the school context: teachers who can accurately identify mental health symptoms are more likely to initiate referral processes, though this capacity is significantly reduced when symptoms are moderate or primarily internalizing, as is common in depression and anxiety [9]. The specific preventive responses available to non-clinical school staff, that is, recognizing signs, providing initial support, and connecting students with appropriate mental health services, are precisely those that depend on symptom and risk factor knowledge rather than on clinical diagnostic skills [10, 11].

Among the most frequent mental disorders affecting adolescents, anxiety and depression stand out due to their prevalence and associated burden. Globally, depression is the fourth leading cause of illness and disability among adolescents aged 15 to 19 years and the fifteenth among those aged 10 to 14 years, while anxiety is the ninth leading cause among those aged 15 to 19 years and the sixth for those aged 10 to 14 years [12]. Anxiety is characterized by excessive worry along with symptoms such as motor restlessness or muscle tension, difficulty maintaining concentration, irritability, or sleep disturbances, causing significant impairment across personal, social, family, educational, and occupational areas of functioning. Depression occurs in varying degrees of severity and tends to be chronic; it is characterized by persistent mood disturbances such as sadness or irritability, decreased interest or pleasure, changes in sleep and appetite, fatigue, and difficulty concentrating, all of which cause clinically significant distress [13].

When untreated, both disorders carry significant long-term consequences: research indicates that many mental disorders in adulthood had their onset in adolescence, coinciding with critical developmental periods that affect biological, psychological, and social functioning [14, 15]. At the population level, an estimated 62,000 adolescents died by suicide globally in 2016, representing the third leading cause of death among those aged 15 to 19 years [12], generating substantial pressure on health systems [16]. Despite this burden, evidence indicates that no efficient measures have been taken to reduce the prevalence of these disorders in adolescents [16], and teachers show limited ability to recognize their symptoms, particularly when these are mild, a deficit closely associated with insufficient knowledge about psychiatric concepts [17].

### The present study

Professionals in educational settings have been identified as having an essential role in recognizing symptoms of depression and anxiety and connecting students with appropriate support as a mechanism for the prevention of suicidal behavior [9]. In Chile, this role is particularly relevant given the magnitude of the problem: national statistics indicate that 16.5% of adolescents present some mental health disorder, with anxiety as the second most prevalent diagnosis (7.4%) and depression the third (7.0%) [18]. The demand for services exceeds current capacity: according to *Defensoría de la Niñez* [19], 14,301 children and adolescents are on a waiting list for outpatient treatment, with anxiety (93%) and depression (92%) among the most frequent presenting problems.

Given that adolescents spend a significant portion of their daily lives in school, educational settings constitute a key context for early identification of mental health problems [11]. However, educational professionals frequently report lacking knowledge, skills, and confidence to fulfill this role effectively [2]. This gap is compounded by the absence of instruments designed to measure knowledge of depression and anxiety within adult educational contexts; existing tools primarily focus on assessing the respondent’s own symptoms rather than their ability to recognize these disorders in others.

This distinction is consequential, as concern expressed by significant individuals who interact with adolescents is among the strongest predictors of help-seeking [20]. Instruments focused on symptom and risk factor knowledge also serve to safeguard the professional boundaries of educational staff, as restricting assessment to these domains prevents the inappropriate expansion of responsibilities into the clinical domain [10, 21].

Therefore, the general objective of the present research was to develop and validate tests assessing knowledge about adolescent depression and anxiety in adults working in the school system. The specific objectives were (a) to create items that measure knowledge about adolescent depression and anxiety, (b) to evaluate the content validity of the items, (c) to estimate the reliability of the instruments, (d) to evaluate the difficulty and discrimination indices of the items, and (e) to evaluate the construct validity of the tests.

## Method

**Fig. 1:**
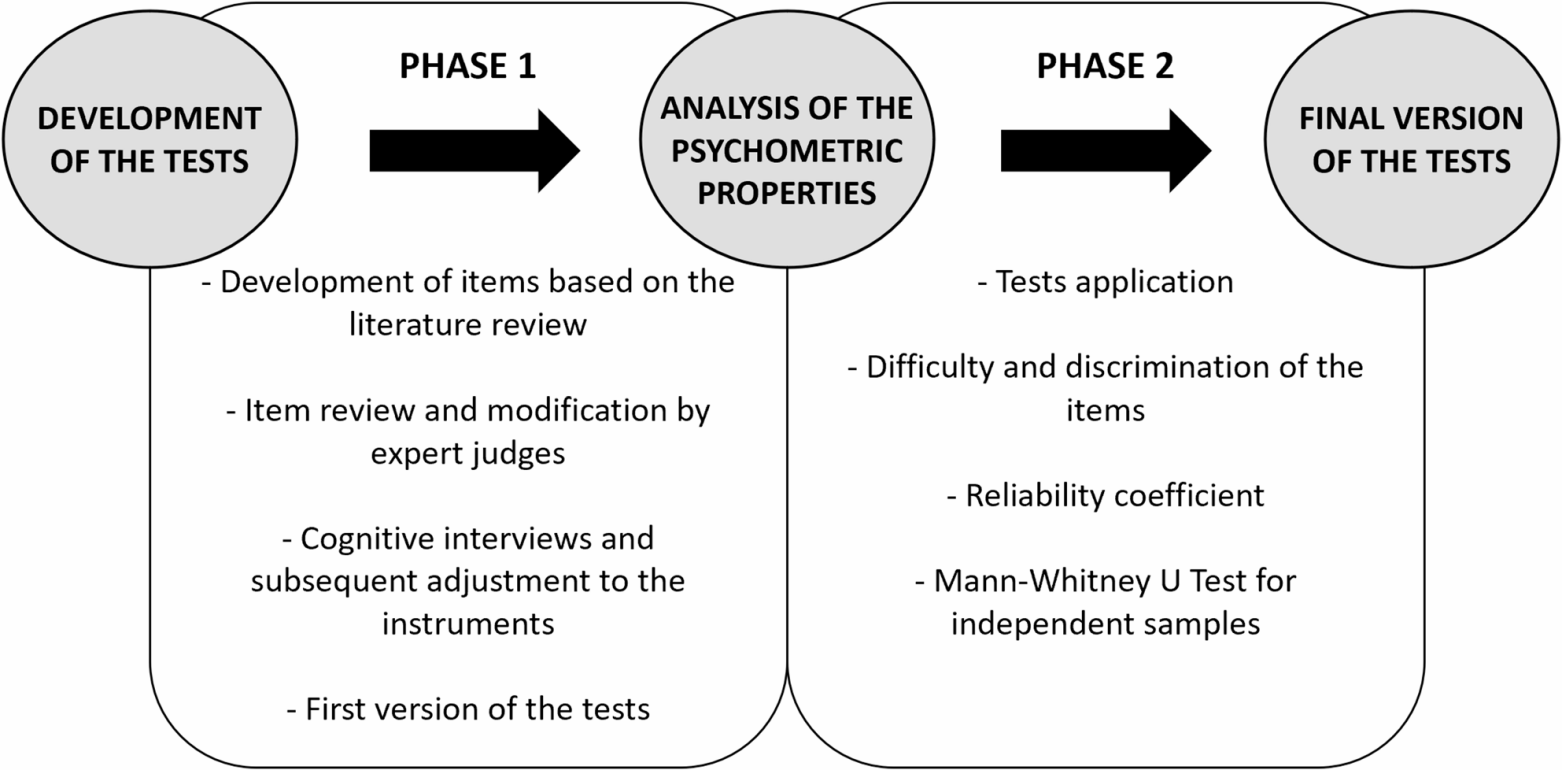
Study execution phases

The research was developed using a quantitative approach. A non-experimental, cross-sectional design was used, and two execution phases were considered (Fig. 1). This design is appropriate for the initial evaluation of psychometric properties, as it allows the assessment of content validity and internal consistency within the study sample, which are the necessary first steps in instrument development. The first phase consisted of the development of the tests: it began with the development of items based on the literature review and the fifth edition of the Diagnostic and Statistical Manual of Mental Disorders (DSM-5), which is the primary classification system for mental health conditions used by psychology and psychiatry professionals [22]. Then these items were reviewed by expert judges and modified, followed by cognitive interviews and subsequent adjustment to the instruments. The second phase consisted of analyzing the psychometric properties: the tests were administered, and the results were used to assess item difficulty and discrimination, the reliability coefficient, and the Mann-Whitney U Test for independent samples.

### Phase 1

#### Participants

A non-probabilistic purposive sampling method was used to select participants. For two months, professionals from different parts of the country were invited by email, in which the purpose of the study and their role in it were explained. The sample consisted of 10 expert judges for content validation, including a teacher in charge of school coexistence, four psychologists, and five child and adolescent psychiatrists, all of whom met the inclusion criterion of at least 5 years of experience. About the professional psychologists, the inclusion criterion was that they had training in dealing with depression and/or anxiety. In this sense, the number of expert judges was determined by the guidelines proposed by Escobar and Cuervo [23], who note that it depends on the level of expertise and the diversity of knowledge, but that around 10 would provide a reliable estimate of the content validity of an instrument.

For the cognitive interviews, invitations were sent to teachers in southern Chile over a three-month period. Finally, sixteen teachers from different areas participated in the cognitive interviews. At the time of the interview, they taught a course from 4th to 5th grade and had at least five years of work experience. In this sense, the total number of cognitive interviews was determined by the suggestion of Willis and Miller [24], who point out that 12 to 15 interviews are needed to meet the saturation and theoretical relevance criteria.

#### Instruments

##### Expert judges’ form

The instruments for data collection corresponded to an expert judges’ form designed in the QuestionPro platform, which was shared with each participant so that they could individually evaluate the test items. This form aimed to assess the relevance, clarity, coherence, and sufficiency of the items incorporated in the test prototype. This worksheet included 76 items, organized in 2 sections (depression and anxiety) of 2 dimensions each (symptoms and risk factors), together with spaces to evaluate the relevance, clarity, and coherence of each item on a scale of 0 to 3 points, where 0 meant the absence of an aspect. Three meant that the item complied satisfactorily with it. In addition, to evaluate sufficiency, at the end of each dimension, we included the question, *“Do you consider that the items are sufficient to measure the dimension? If not, which items would you suggest?”*

##### Cognitive interviews

For the cognitive interviews, a self-developed interview guide was used to (a) learn about the participants’ response processes, (b) evaluate the level of relevance of the items, and (c) identify whether any of the cognitive problems exist when applying the tests, such as Inconsistency between the question and the response options and Confusing, inadequate, non-exclusive or missing response categories.

#### Procedure

 This phase consisted of a review of the DSM-5 and the literature on the constructs of adolescent depression and anxiety. Based on the information gathered, a content analysis was conducted to identify the most relevant aspects, since to establish a possible universe of items, it is necessary to have an adequate conceptualization and operationalization of the construct, i.e., the dimensions to be measured and their indicators from which the items will be made must be specified beforehand [23].

To guide item development and ensure balanced coverage of the content domains, a test blueprint (table of specifications) was elaborated following the recommendations for test construction proposed in the Standards for Educational and Psychological Testing [25]. This scheme allowed the systematic organization of items according to the theoretical dimensions and indicators identified in the literature. It was possible to find mainly two dimensions to be included in the depression and anxiety constructs: symptoms and risk factors. Subsequently, the items were written as statements and organized into two sections corresponding to depression and anxiety. In contrast, each section was divided into the two dimensions indicated.

Subsequently, the items were constructed using a True/False/Don’t know response format. This format facilitates comprehension, reduces response time, and allows respondents to explicitly indicate lack of knowledge. From a psychometric perspective, the inclusion of a “Don’t know” option reduces random guessing and yields more accurate estimates of respondents’ knowledge in factual assessments [26].

“Don’t know” responses were coded as incorrect. This decision treats the absence of knowledge and incorrect knowledge as equivalent for scoring purposes, which is consistent with knowledge tests focused on the identification of correct information. Coding “Don’t know” as incorrect also prevents inflation of scores due to guessing and supports a more conservative estimation of knowledge levels.

The dichotomous structure of the response format increases the probability of correct responses by chance and constrains response variability, which directly affects item discrimination [27]. At the same time, the inclusion of the “Don’t know” option partially offsets guessing effects by providing a non-forced alternative to True/False responses.

Subsequently, expert judges conducted content validity following Escobar and Cuervo’s theoretical proposal [23]. The results showed that the concordance among judges was calculated using Aiken’s V coefficient and the Excel program.

In this same phase, individual and virtual interviews were conducted through the Zoom platform, which were recorded with the interviewees’ consent and then transcribed. Before each interview, the teachers were asked to answer the tests. The comprehension of the questions and the usability of the participants’ processing and responses to the instruments were examined. Then, based on the comments, some items’ necessary semantic adjustments were made, culminating in the first version of the tests that the teachers had prepared.

### Phase 2

#### Participants

In the second phase, corresponding to the application of the tests, these were answered by professionals from educational establishments in southern Chile who were invited via email sent to their respective schools. Invitations were sent to 32 educational establishments over a five-month period, between August and December 2023, requesting voluntary participation from educational professionals working in those institutions. A total of 304 valid responses from professionals belonging to these establishments were obtained.

Psychologists’ responses were excluded to preserve the instrument’s conceptual focus on non-clinical educational professionals, such as teachers and other school staff, whose mental health training is typically more limited and differs substantially from that of clinically trained professionals. Including psychologists could have introduced systematic differences in responses due to their specialized knowledge and professional expertise in mental health, potentially affecting the interpretation of the instrument and the comparability of results within the target population [28]. Additionally, it is important to acknowledge the possibility of self-selection bias, since participation in the study was voluntary, and professionals with a greater interest or sensitivity toward mental health issues may have been more likely to respond, a phenomenon frequently observed in voluntary survey research and which may influence the representativeness of the sample [29].

In addition, we considered only the responses of teachers who taught from 5th grade of primary school through 4th grade of secondary school (*5° básico* to *4° medio* in the Chilean educational system), had at least five years of experience, and had completed the entire test. Thus, the responses of 281 professionals from municipal (*n* = 179) and private subsidized (*n* = 102) educational establishments in southern Chile were included. This sample size considers representation of at least 10 participants per item for each scale.

##### Sociodemographic questionnaire

In the second phase of the study, together with the application of the tests developed, a sociodemographic questionnaire was included that collected information from the participants, such as age, gender, profession, years of work experience, current position, and attendance to training on depression and/or anxiety.

#### Procedure

Invitations to participate were sent via email to 32 educational establishments in southern Chile over a five-month period, from August to December 2023, requesting voluntary participation from professionals working at those institutions. The survey was administered virtually, and prior to responding, participants were presented with an informed consent form. Only those who agreed to its terms could proceed with the survey.

### Overall data analysis

In the first phase, the quality of the interviews was examined through a content analysis of the questions posed to participants. Items were grouped into analytically derived categories, and content validity was evaluated by a panel of expert judges using Aiken’s V coefficient. This coefficient quantifies the degree of agreement among experts regarding the relevance and adequacy of each item, yielding standardized values between 0 and 1, where higher scores indicate greater consensus and perceived item quality. In line with commonly accepted psychometric criteria, values equal to or above 0.70 were considered indicative of adequate content validity. This procedure allowed the identification of items with adequate content representation, as well as those requiring reformulation or elimination based on expert judgment [23].

In the second phase, an item difficulty analysis was conducted. For each item, a difficulty index (*p*) was calculated as the proportion of respondents who answered the item correctly. Values closer to 1 indicate easier items, whereas values closer to 0 indicate more difficult items. Items with extreme difficulty values provide limited psychometric information, as they show reduced capacity to discriminate between individuals with different levels of the underlying construct. Consequently, items with intermediate difficulty levels were considered more informative for the measurement purposes of the scale.

A discrimination analysis was conducted using two complementary indicators to evaluate each item’s ability to differentiate between respondents with different levels of the construct. First, a group-based discrimination index was calculated by comparing the performance of respondents with high and low overall test scores. This index reflects the extent to which an item distinguishes between individuals at different levels of the construct, with higher values indicating greater discriminative capacity. In accordance with classical test theory recommendations, items with discrimination index values of 0.39 or higher were considered adequate and retained, whereas items with lower values were considered for potential removal [30].

Second, the discrimination coefficient, operationalized as the item–total point-biserial correlation, was examined to assess the association between each item score and the total test score. This coefficient indicates the degree to which an item consistently contributes to the overall measurement of the construct. Consistent with established psychometric guidelines, values of 0.30 or higher were considered indicative of acceptable item discrimination [26, 31, 32]. The initial set of item-level analyses was conducted using Microsoft Excel.

In addition, the Kuder-Richardson reliability coefficient was calculated for each dimension using IBM SPSS Statistics software v26 [33]. Coefficients equal to or above 0.70 were considered indicative of adequate internal consistency, in line with established criteria for knowledge-type instruments [32]. The Mann-Whitney U test for independent samples was conducted using JASP [34] to contribute evidence of construct validity, under the hypothesis that participants previously trained in depression and/or anxiety would obtain higher scores than those without such training, given that even brief training sessions have been shown to increase knowledge on these topics [35]. Effect sizes were estimated using the rank-biserial correlation coefficient, a measure appropriate for nonparametric comparisons between two independent groups, and interpreted according to the conventional criteria for correlation-based effect sizes proposed by Cohen [36], where values around 0.10 indicate small effects, values around 0.30 indicate medium effects, and values around 0.50 indicate large effects.

## Results

### Preparation of items

In the first phase of the study, corresponding to the literature review for elaborating the items, 76 True or False items were obtained, where all statements were true. The Depression and Anxiety tests were divided into two dimensions: symptoms and risk factors. Some of the items were *“Persistent loss of interest in activities is a symptom of depression”*,* “A family history of depression is a risk factor”*,* “Someone with anxiety may have trouble breathing”*, and *“Being under constant stress is a risk factor for anxiety”.*

### Analysis of the qualities of the interviews

The main results from the cognitive interviews fell into 2 categories. The first is the category *Response options*, where the participants suggested including the option *Don’t know*, since, when asked if the response options facilitated the way they answered the tests, they mainly obtained responses such as the following: “The truth, because as I was saying, there were things I did not know and the I don’t know option is also valid, but since there is no I don’t know, I am obliged to choose false. So, it is like choosing something and to be pigeonholed into something, but it doesn’t represent what I want”.

The second category is Duration of the tests, where the participants considered the duration appropriate. When asked if they felt fatigued at any time due to the time it took them to answer the tests, they indicated that they did not and considered the number of items adequate.

### Aiken V coefficient

About the results obtained with the validation of content by expert judges by calculating the Aiken V coefficient and the average for each item, it was determined that 51 items had a high concordance index (V = > 0.8). Some of the retained items were *“A person with depression feels sad almost every day”*,* “A family history of depression is a risk factor”*,* “Anxiety can be a symptom of other mental disorders”*, and *“A family history of anxiety is a risk factor”*. While 25 items did not have a high concordance index (value < 0.7) or a high concordance between judges, the criterion indicated that the item was inadequate, so they were discarded. Some of the discarded items were *“Decreased class attendance is a symptom of depression”*,* “An unhealthy diet is a risk factor for depression”*,* “Tremors are a symptom of anxiety”*, and *“A reduced ability to concentrate is a risk factor for anxiety”.*

The most relevant comments from the expert judges pointed out that absolutism in the wording of the items should be avoided, and recommended reducing the length of the tests by combining some items.

### Discrimination index

This analysis aims to eliminate less effective items in discriminating between those with high and low overall scores. In this sense, an index > 0.39 is excellent, so the item should be retained. On the other hand, an item with an index < 0.38 is discarded.

### Discrimination coefficient

This analysis aims to show the correlation between the score of each item and the total score. Items with an index higher than 0.30 were retained (Tables 1, 2 and 3, and 4).

**Table 1:** Depression scale: symptoms (*n* = 20). Coefficient KR-20 = 0.864 (95% CI 0.823–0.906)

Item number	Discrimination index	Discrimination coefficient	Difficulty index
1	0.471	0.517	0.88
2*	0.483	0.237	0.57
3	0.460	0.503	0.89
4	0.644	0.368	0.68
5	0.586	0.553	0.83
6	0.483	0.625	0.90
7	0.586	0.517	0.81
8	0.690	0.498	0.74
9	0.575	0.419	0.77
10	0.494	0.473	0.86
11*	0.391	0.523	0.92
12	0.471	0.494	0.88
13	0.655	0.508	0.79
14	0.460	0.626	0.91
15	0.517	0.427	0.82
16*	0.391	0.436	0.92
17	0.701	0.567	0.78
18	0.770	0.406	0.58
19	0.529	0.519	0.85
20	0.805	0.482	0.60

The asterisk (*) indicates items that were discarded

**Table 2:** Depression scale: risk factors (*n* = 10). Coefficient KR-20 = 0.742 (95% CI 0.680–0.803)

Item number	Discrimination index	Discrimination coefficient	Difficulty index
1	0.638	0.357	0.67
2	0.676	0.336	0.59
3*	0.467	0.317	0.26
4	0.457	0.463	0.88
5	0.590	0.374	0.70
6	0.667	0.469	0.72
7	0.486	0.479	0.86
8*	0.362	0.464	0.93
9	0.429	0.549	0.91
10	0.552	0.494	0.83
11	0.638	0.402	0.64
12	0.648	0.351	0.41

The asterisk (*) indicates items that were discarded

**Table 3:** Anxiety scale: symptoms (*n* = 12). Coefficient KR-20 = 0.873 (95% CI 0.838–0.907)

Item number	Discrimination index	Discrimination coefficient	Difficulty index
1	0.570	0.390	0.73
2	0.672	0.402	0.51
3	0.602	0.647	0.83
4	0.703	0.661	0.77
5	0.563	0.679	0.85
6	0.578	0.653	0.84
7	0.641	0.421	0.67
8	0.734	0.496	0.70
9	0.547	0.603	0.82
10	0.500	0.687	0.89
11	0.531	0.668	0.87
12	0.523	0.663	0.87

**Table 4:** Anxiety scale: risk factors (*n* = 7). Coefficient KR-20 = 0.846 (95% CI 0.805–0.887)

Item number	Discrimination index	Discrimination coefficient	Difficulty index
1	0.856	0.494	0.63
2	0.808	0.676	0.82
3	0.788	0.684	0.83
4	0.781	0.708	0.84
5	0.733	0.629	0.88
6	0.897	0.586	0.73
7*	0.459	0.287	0.31

The asterisk (*) indicates the item that was deleted

Among retained items, discrimination indices ranged from 0.460 to 0.805 for depression symptoms, 0.429 to 0.676 for depression risk factors, 0.500 to 0.734 for anxiety symptoms, and 0.733 to 0.897 for anxiety risk factors. Discrimination coefficients ranged from 0.368 to 0.626, 0.336 to 0.549, 0.390 to 0.687, and 0.494 to 0.708 for the same dimensions, respectively, with all retained items surpassing the established thresholds.

### Difficulty index

The difficulty index represents the percentage of people who answered the item correctly. Those answered correctly (or incorrectly) by many people are unlikely to discriminate among examinees and, therefore, were candidates for elimination. For example, a value of 0.95 indicates that most people respond correctly to the item and that the item provides little helpful information and may detract from the psychometric properties of the scale, just as a too-low value would.

Based on the evaluation of the results obtained for each item, it was determined that for the symptoms dimension of the depression scale, item 2 *(Spanish: Una persona con depresión tiene un estado de tristeza casi todos los días / English: A person with depression has a state of sadness almost every day)*, 11 *(Spanish: Alguien con depresión puede intentar suicidarse / English: Someone with depression may attempt suicide)* and 16 *(Spanish: Los síntomas de depresión pueden variar entre una persona y otra / English: The symptoms of depression may vary from one person to another)* would be discarded. This is because, besides not having adequate indices, their content was considered irrelevant since other items provide similar information and have relevant index.

For the risk factors dimension of the anxiety scale, only item 7 *(Spanish: Los síntomas relacionados con la ansiedad pueden deberse a enfermedades médicas*,* como de la glándula tiroides / English: Anxiety-related symptoms may be due to medical illnesses*,* such as thyroid gland)* was eliminated, as it was not considered relevant to the dimension.

Among retained items, difficulty indices ranged from 0.58 to 0.91 for depression symptoms, 0.41 to 0.91 for depression risk factors, 0.51 to 0.89 for anxiety symptoms, and 0.63 to 0.88 for anxiety risk factors.

### Reliability

The Kuder-Richardson reliability coefficient was analyzed for each scale. In this sense, the depression scale’s coefficient for the symptoms dimension was 0.864, and for the risk factors dimension, 0.742. The anxiety scale’s coefficient for the symptoms dimension was 0.873, and for the risk factors dimension, 0.846. These results suggest adequate internal consistency in the two tests.

In conclusion, after the analyses, it was determined that the depression scale would have 27 items (17 in the symptom dimension and 10 in risk factors), and the anxiety scale would have 18 items (12 in the symptom dimension and 6 in risk factors). A detailed summary of the changes at each stage is shown in Table 5.

**Table 5:** Items for each dimension throughout the stages of the study

	Depression: Symptoms	Depression: Risk factors	Anxiety: Symptoms	Anxiety: Risk factors
Initial items elaborated based on the literature review	29	17	16	14
Items discarded after the expert judges’ revision	9	5	4	7
Items discarded after cognitive interviews	0	0	0	0
Items discarded after test application	3	2	0	1
Final items	17	10	12	6

### Mann-Whitney U Test

Finally, regarding the results of the Mann-Whitney U test for independent samples comparing participants who had attended training on depression and/or anxiety with those who had not, trained participants obtained significantly higher scores in most dimensions (Table 6). Significant differences were observed in the symptom dimensions of both depression (*r* = 0.219, *p* = 0.006) and anxiety (*r* = 0.254, *p* = 0.001), as well as in the anxiety risk factors dimension (*r* = 0.215, *p* = 0.005). Although higher scores were also observed among trained participants for depression risk factors, this difference did not reach statistical significance (*r* = 0.147, *p* = 0.068). Across all dimensions, effect sizes were small according to conventional criteria [36], indicating that while the direction of differences consistently favored trained participants, the magnitude of these differences was modest.

**Table 6:** Comparison of means between those who have attended training and those who have not

Tests	Groups	Correct answers (Mean)	*p* value	Effect size Rank-biserial correlation [95% CI]
Depression: symptoms dimension (*n* = 17)	1 (*n* = 66) 2 (*n* = 215)	14.379 13.316	0.006	0.219 [0.063–0.365]
Depression: risk factors dimension (*n* = 10)	1 (*n* = 66) 2 (*n* = 215)	7.697 7.065	0.068	0.147 [0.012–0.298]
Anxiety: symptoms dimension (*n* = 12)	1 (*n* = 66) 2 (*n* = 215)	10.182 9.093	0.001	0.254 [0.100–0.397]
Anxiety: risk factors dimension (*n* = 6)	1 (*n* = 66) 2 (*n* = 215)	5.242 4.577	0.005	0.215 [0.058–0.361]

Group 1 = They have attended training. Group 2 = They have not attended training

## Discussion

This study provides evidence of the reliability and validity of tests of knowledge about adolescent depression and anxiety to be answered by adults in the school system. Together, these instruments contain 45 items with True - False - Don’t Know response options to assess knowledge of adolescent depression and anxiety among adults in the school system. The scales take approximately 10 min to complete, covering symptom recognition and risk factors. They are designed for use with education professionals (e.g. teachers, principals).

In this sense, the analysis of the psychometric properties of the tests suggests that they have adequate reliability, content validity, and construct validity [25, 26, 30, 32]. In relation to the latter, it was expected that those who had training on depression and/or anxiety would obtain more correct answers compared to those who did not, which turned out to be significantly evident in the tests.

On the other hand, concerning the items discarded from the analysis of the psychometric properties, it is considered that their elimination was pertinent since they were not relevant for the corresponding dimensions, according to the expert judges. For example, the three items discarded in the symptoms dimension of the depression scale had similar content to other items of the same dimension. Also, item 7 (*Anxiety-related symptoms may be due to medical illnesses*,* such as thyroid gland*), which was eliminated from the anxiety risk factors dimension, because its coefficient of discrimination was low due to its high difficulty, so most people answered it incorrectly.

It is important to point out that the tests are not diagnostic tools, nor are they intended for health professionals; instead, the test is focused on recognizing symptoms, warning signs, and risk factors by education professionals. For this reason, dimensions found in other mental health knowledge instruments, such as assessment and diagnosis, course, impact on life, care, and treatment, were not included. Rather, it contains representative items that would reflect knowledge of symptoms, warning signs, and risk factors on the constructs of the study.

This decision was based primarily on conceptual and ethical considerations. Conceptually, knowledge of symptoms and risk factors is directly related to the preventive and early detection role expected of teachers and other adults in the school setting, while aspects related to care and treatment fall within the realm of clinical intervention and exceed the professional responsibilities typically assigned to education professionals [2, 21]. From an ethical perspective, including content related to care and treatment could contribute to role confusion and an implicit expectation that teachers make clinical decisions, which may be inappropriate given their professional training and responsibilities [21]. Therefore, the results should be interpreted within the scope of this conceptual framework, and future research could explore complementary approaches focused on referral processes and collaboration with mental health services.

In applied terms, scores can inform decisions about training needs at the individual, classroom, or institutional level, providing a concrete and systematic basis for designing, prioritizing, or evaluating professional development initiatives focused on mental health in school settings. This is particularly relevant in contexts such as Chile, where demand for mental health services among adolescents exceeds current capacity and where non-clinical school staff constitute a first line of contact with students showing early signs of distress [18]. By identifying specific areas of knowledge deficit, the instruments can contribute to more targeted and efficient use of training resources, rather than relying on generic or undifferentiated professional development programs. These uses are consistent with the psychometric evidence reported here, which supports the instruments’ internal consistency, content validity, and sensitivity to differences associated with prior training experience.

The extent to which improvements in knowledge scores translate into changes in actual professional practices has not been examined in this study and cannot be assumed from the psychometric evidence alone. The relationship between knowledge and behavior is not automatic, and educational professionals may increase their scores on a knowledge measure without this necessarily producing observable changes in how they identify or respond to students showing signs of depression or anxiety. Establishing whether this gap can be bridged requires validation approaches that go beyond psychometric adequacy, and this remains a necessary step before broader claims about the instruments’ practical utility can be made. Nevertheless, having a psychometrically sound starting point is a prerequisite for this type of validation, and the evidence reported here provides the foundation from which predictive and intervention-based studies can be built, with the potential to substantially strengthen the instruments’ contribution to school-based mental health practice.

Several limitations of the present study should be acknowledged. First, the sample was non-probabilistic and regionally bounded, drawn exclusively from educational establishments in southern Chile, which constrains the generalizability of the findings to other regional, national, or cultural contexts. In this regard, a potential selection bias must be acknowledged; the most likely direction of this bias points toward an overestimation of general knowledge about adolescent depression and anxiety, as it is plausible that professionals with greater interest or prior training in mental health were more motivated to respond. Nonetheless, since this bias may vary across specific test dimensions or sample subgroups, and information on non-respondents is unavailable, the exact magnitude of this distortion is difficult to determine.

Second, psychometric analyses were conducted under Classical Test Theory, which was appropriate given the sample size and the exploratory nature of the study, but precluded more advanced approaches such as Item Response Theory, which would allow simultaneous estimation of item difficulty and discrimination at the individual level, or measurement invariance testing across subgroups such as teaching role or prior training experience.

Third, the dichotomous response format, while practical for rapid administration and scoring in applied educational settings, carries specific psychometric limitations in the context of knowledge assessment. True/False items are particularly susceptible to guessing, given that the probability of a correct response by chance alone is 0.50, which inflates observed scores and attenuates item discrimination indices, reducing the instrument’s capacity to differentiate between respondents with genuine knowledge and those responding randomly. This is especially relevant when the target construct involves nuanced clinical content, such as the differentiation of depressive symptoms from normative adolescent behavior, where partial or uncertain knowledge may lead professionals to guess rather than endorse a “Don’t know” response. Although the inclusion of this third option was intended to mitigate forced responding, it introduces scoring ambiguity in the sense that, depending on how “Don’t know” responses are treated in analyses (whether as incorrect, as missing, or as a neutral category), reliability estimates and item difficulty parameters may vary substantially, complicating interpretation and cross-study comparisons.

Future research should prioritize replication in larger, more diverse, and probabilistically selected samples, ideally through multi-site recruitment strategies that include different regions and educational contexts, in order to establish more robust generalizability evidence. Once larger samples are available, Item Response Theory analyses and measurement invariance testing across relevant subgroups, such as teaching role, educational level, or prior training experience, would provide more precise information about item functioning and the comparability of scores across groups. Regarding the response format, future versions of the instruments could incorporate scenario-based items that allow for greater contextualization of symptoms while maintaining feasibility in school settings. Finally, longitudinal and intervention-based designs would be particularly valuable to examine whether knowledge gains, as measured by these instruments, are associated with improved recognition practices, referral behavior, or engagement in preventive actions within school contexts.

In conclusion, this study developed and provided initial psychometric evidence for two instruments assessing knowledge of symptoms and risk factors of adolescent depression and anxiety among adults in educational contexts. Both instruments demonstrated adequate internal consistency and content validity, and group comparisons supported their sensitivity to differences in prior training. As a first version, they offer an initial psychometric base that requires further refinement and testing in future studies. These findings position them as viable tools for identifying knowledge gaps and informing training decisions in school settings, pending replication in larger, more diverse, and probabilistically selected samples. Given the scarcity of instruments specifically designed to assess this knowledge domain in educational contexts, the present study addresses a documented gap in the field and provides a foundation for subsequent validation efforts.

## Supplementary Information

Supplementary Material 1.

## Data Availability

The tests developed in this study are provided as supplementary material, facilitating its utilization for future investigations. The dataset produced and examined in the present research will be accessible upon reasonable request from the corresponding author (MB).
