# Supplementary material for: Knowledge tests on adolescent depression and anxiety: a measure for adults in the Chilean school system
Source: BMC Psychol. 2026 Jun 20;14:1090. doi: 10.1186/s40359-026-04674-4 (PMC13397628; doi:10.1186/s40359-026-04674-4)
Supplement: Supplementary file 1 — Supplementary Material 1. [file 40359_2026_4674_MOESM1_ESM.docx]

Supplementary material “Final version of tests (English)”

**TEST I: KNOWLEDGE OF DEPRESSION**

DIMENSION I: SYMPTOMS

| For each of the following statements choose ***True (T)***, ***False (F)*** or ***Don’t know*** by placing an **X** in the appropriate box. | **T** | **F** | **Don’t know** |
| --- | --- | --- | --- |
| 1. Persistent loss of interest in daily activities is a symptom of depression |  |  |  |
| 2. It is common for a person with depression to feel “sad”, “empty”, “hopeless” |  |  |  |
| 3. In people with depression, mood may be irritable |  |  |  |
| 4. Daytime drowsiness or insomnia may be symptoms of depression |  |  |  |
| 5. Someone with depression can feel a loss of energy |  |  |  |
| 6. Someone with depression can feel useless most of the time |  |  |  |
| 7. Someone with depression could feel guilty most of the time |  |  |  |
| 8. Depression could reduce decision-making ability |  |  |  |
| 9. Someone with depression may have recurrent thoughts of death |  |  |  |
| 10. Loss of interest in previously enjoyable activities may be a sign of depression |  |  |  |
| 11. Depression could manifest itself in interpersonal problems |  |  |  |
| 12. Someone with depression may think they are unable to cope with their sadness |  |  |  |
| 13. A person with depression may suffer from another pathology, for example, anxiety |  |  |  |
| 14. Decreased appetite could be a symptom of depression |  |  |  |
| 15. Increased appetite may be a symptom of depression |  |  |  |
| 16. Depression could decrease the ability to concentrate |  |  |  |
| 17. Learning difficulties could be a symptom of depression |  |  |  |

DIMENSION II: RISK FACTORS

| For each of the following statements choose ***True (T)***, ***False (F)*** or ***Don’t know*** by placing an **X** in the appropriate box. | **T** | **F** | **Don’t know** |
| --- | --- | --- | --- |
| 1. Family history of mood disorders is a risk factor for depression |  |  |  |
| 2. Family history of substance use is a risk factor for depression |  |  |  |
| 3. Bullying is a risk factor for depression |  |  |  |
| 4. Poor academic performance is a risk factor for depression |  |  |  |
| 5. Alcohol consumption is a risk factor for depression |  |  |  |
| 6. Depression at an early age and without adequate treatment enables its persistence in adulthood. |  |  |  |
| 7. Domestic violence is a risk factor for depression |  |  |  |
| 8. Perceived lack of support is a risk factor for depression |  |  |  |
| 9. Depression rates increase from the onset of puberty onwards |  |  |  |
| 10. Symptoms related to depression may be due to medical conditions, such as thyroid disease |  |  |  |

**TEST II: KNOWLEDGE OF ANXIETY**

DIMENSION I: SYMPTOMS

| For each of the following statements choose ***True (T)***, ***False (F)*** or ***Don’t know*** by placing an **X** in the appropriate box. | **T** | **F** | **Don’t know** |
| --- | --- | --- | --- |
| 1. Anxiety could be a symptom of other mental health conditions |  |  |  |
| 2. Someone with anxiety could have dry mouth |  |  |  |
| 3. Someone with anxiety could have trouble breathing in certain situations |  |  |  |
| 4. Excessive sweating could be a physical symptom of anxiety |  |  |  |
| 5. Someone with anxiety could have an intense fear of public speaking |  |  |  |
| 6. Feeling agitated and nervous could be a symptom of anxiety |  |  |  |
| 7. Someone with anxiety could feel fatigue |  |  |  |
| 8. Muscle tension could be a symptom of anxiety |  |  |  |
| 9. Someone with anxiety could have difficulty concentrating |  |  |  |
| 10. Someone with anxiety could feel excessive worry |  |  |  |
| 11. Someone with anxiety could have difficulty falling asleep |  |  |  |
| 12. Someone with anxiety could have constant sleep interruptions |  |  |  |

DIMENSION II: RISK FACTORS

| For each of the following statements choose ***True (T)***, ***False (F)*** or ***Don’t know*** by placing an **X** in the appropriate box. | **T** | **F** | **Don’t know** |
| --- | --- | --- | --- |
| 1. Family history of mental health disorders may be a risk factor for anxiety |  |  |  |
| 2. Having experienced a traumatic event can be a risk factor for anxiety |  |  |  |
| 3. Sexual abuse can be a risk factor for anxiety |  |  |  |
| 4. Domestic violence can be a risk factor for anxiety |  |  |  |
| 5. Being under constant stress can be a risk factor for anxiety |  |  |  |
| 6. Low self-esteem can be a risk factor for anxiety |  |  |  |
